# Prospective Study of Chikungunya Virus Acute Infection in the Island of La Réunion during the 2005–2006 Outbreak

**DOI:** 10.1371/journal.pone.0007603

**Published:** 2009-10-28

**Authors:** Frederik Staikowsky, François Talarmin, Philippe Grivard, Abdel Souab, Isabelle Schuffenecker, Karin Le Roux, Marc Lecuit, Alain Michault

**Affiliations:** 1 Emergency Department, Pôle des Spécialités de l'Urgence, Centre Hospitalier Régional de La Réunion, Groupe Hospitalier Sud Réunion, Saint Pierre, La Réunion, France; 2 Department of Microbiology, Pôle de biologie, Centre Hospitalier Régional de La Réunion, Groupe Hospitalier Sud Réunion, Saint Pierre, La Réunion, France; 3 National Reference Center for Arboviruses, Institut Pasteur, Lyon, France; 4 Institut Pasteur, Microbes and host barriers Group, Paris, France; 5 Inserm, Avenir U604, Paris, France; 6 Department of Infectious Diseases and Tropical Medicine, Paris Descartes University, Necker-Pasteur Center for Infectious Diseases, Hôpital Necker-Enfants Malades, Assistance Publique-Hôpitaux de Paris, Paris, France; Institut Pasteur, France

## Abstract

**Background:**

Chikungunya virus (CHIKV) is a recently re-emerged arthropod borne virus responsible for a massive outbreak in the Indian Ocean and India, and extended to Southeast Asia as well as Italy. CHIKV has adapted to *Aedes albopictus*, an anthropophilic mosquito species widely distributed in Asia, Europe, Africa and America. Our objective was to determine the clinical and biological features of patients at the acute phase of CHIKV infection.

**Methods and Findings:**

A prospective study enrolled 274 consecutive patients with febrile arthralgia recorded at the Emergency Department of the Groupe Hospitalier Sud-Réunion between March and May 2006. Three groups were defined: one group of 180 viremic patients (positive CHIKV RT-PCR), one group of 34 patients with acute post-viremic infection (negative CHIKV RT-PCR, positive anti-CHIKV IgM and negative IgG), and one group of 46 uninfected patients (negative CHIKV RT-PCR, anti-CHIKV IgM and IgG). Bivariate analyses of clinical and biological features between groups were performed. Patients with CHIKV viremia presented typically with asymmetrical bilateral polyarthralgia (96.5%) affecting the lower (98%) and small joints (74.8%), as well as asthenia (88.6%), headache (70%), digestive trouble (63.3%), myalgia (59%), exanthems (47.8%), conjunctival hyperhemia (23%) and adenopathy (8.9%). Vertigo, cutaneous dysesthesia, pharyngitis and haemorrhages were seldom observed. So far unreported symptoms such as chondrocostal arthralgia (20%), entesopathies (1.6%), talalgia (14%) were also noted. Prurit was less frequent during the viremic than post-viremic phase (13.9% vs. 41.2%; p<0.001), whereas lymphopenia was more frequent (87.6% vs. 39.4%; p<0.001). Others biological abnormalities included leukopenia (38.3%), thrombocytopenia (37.3%), increased ASAT and ALAT blood levels (31.6 and 7.3%, respectively) and hypocalcemia (38.7%). Lymphopenia <1,000/mm^3^ was very closely associated with viremic patients (Yule coefficient 0.82, positive predictive value 92.3%). Age under 65 was associated with a benign course, as no patients younger than 65 had to be hospitalized (Yule coefficient 0.78).

**Conclusions:**

The diagnosis of CHIKV infection in acute phase is based on commonly accepted clinical criteria (fever and arthralgia), however clinical and biological diffrences exist in acute phase depending on whether or not the patient is within the viremic phase of the infection.

## Introduction

Chikungunya virus (CHIKV) is a positive strand RNA enveloped alphavirus belonging to the *Togaviridae* family. CHIKV was first isolated by R.W. Ross [1] in the Newala district of Tanzania and is responsible for acute febrile arthralgia in human. Since 1952, several outbreaks of Chikungunya fever have been reported in Sub-Saharan Africa, Asia (India, Pakistan, South-East Asia, Philippines), and the islands of the Pacific Ocean [2]. CHIKV has emerged in the South-Western Indian Ocean region in the beginning of 2005. Between March 2005 and September 2006, 38.2% of the 785,000 inhabitants of La Réunion Island [3], [4] were infected. This is the first outbreak recorded on this territory.

By including all patients referred to the Emergency Department (ED) with febrile arthralgia, the aim of this study was to describe prospectively the clinical and biological features of acute CHIKV infection and identify features to help differentiate CHIKV infection from other conditions. Using CHIKV-specific reverse transcription and polymerase chain reaction (RT-PCR) and serology, we also compared patients in the acute viremic phase and those in the post-viremic phase. We also investigated co-infections. Finally, we also identified clinical and biological markers for severity in acute CHIKV infection patients by comparing hospitalized patients to those who were not.

## Methods

The prospective study was conducted in the ED of the Groupe Hospitalier Sud Réunion (GHSR) Regional Hospital. This hospital's standards are similar to those found in mainland France. It receives people living in the Southern part of the Island of La Réunion (350,000 inhabitants). The hospital has 1,154 beds and 316 physicians. The ED receives 41,000 patients annually; with adults being treated for medical and surgical conditions, and children for surgical conditions only.

We enrolled all patients over 15 years of age referred for febrile arthralgia at the ED between March 1^st^ 2006 and May 31^st^ 2006. A clinical examination, a blood sampling and a questionnaire were completed for all included subjects as the time of admission at the ED. This was the only time those patients were examined and interrogated for the purpose of this study.

The questionnaire was written after a 2-months observation period (January to February 2006, when the epidemic was at its peak, with an incidence of 45,000 cases per week). The questionnaire was created and validated after the medical file analysis of 1,030 consecutive patients referred to the ED with febrile arthralgia evocative of Chikungunya. The data collected included age, gender, medical history (co-morbidity, recent travels abroad), symptoms and date of their onset (1^st^ day), the need for hospitalization and biological parameters.

Oral consent was obtained from each patient or a first-degree relative, as the investigations were carried out under the standard care procedure for this poorly characterized disease, in accordance with the recommendations of the Committee for Clinical Research of the GHSR. In France, written consent is mandatory only if the medical treatments or the products used are not standard for the diagnosis, treatment, or monitoring (*art. 88-II, law 2004–806, Journal Officiel, 08/11/2004; art. 31-I, law 2006–450, Journal Officiel, 04/19/2006*). The information was given in French or in Creole with the help of a translator when necessary.

Chikungunya is known to evolve in successive phases: first, a viremic acute phase that lasts around 5 days, followed by a post-viremic acute phase defined by a negative CHIKV RT-PCR, presence of anti-CHIKV IgM antibodies but absence of IgG, that lasts about a week before the appearance of anti CHIKV IgG [5]. Please note that this chronology only applies to patients originating from area where CHIKV has not circulated previously, as it is the case in La Réunion. CHIKV-specific IgM and IgG antibodies were detected by capture enzyme-linked immuno-sorbent assay (ELISA) [6] and CHIKV-specific IgG antibodies by ELISA developed at the French National Reference Centre for Arbovirus (Institut Pasteur, Lyon, France) and automated with an EtiMax 3000 apparatus (DiaSorin®, Italy). The cut-off values for IgGs and IgMs were determined from a series of 30 negative sera collected in La Réunion before the epidemic. A one-step TaqMan® real time quantitative RT-PCR was performed in serum samples using the Light Cycler 2.0® (Roche Diagnostics). The assay sensitivity for serum specimen was 350 copies per mL [7]. The patients enrolled in the study were separated into two groups: Group A, patients with Chikungunya, Group B, patients without Chikungunya (negative RT-PCR and negative IgM anti CHIKV). In Group A the patients were in the acute infection phase (RT-PCR + or CHIKV Specific IgM + and IgG -). Within Group A, two subgroups were defined: Group A1, patients with viremia (RT-PCR+) and Group A2, patients without viremia. Whether the patients in the acute infection phase (Group A) had been hospitalized or not was used as a severity criterion.

Patients with positive anti-CHIKV IgG (*i.e.* that had been infected with CHIKV previously) were excluded from the study.

The study subjects were tested for other agents susceptible of causing fever and arthralgia. These include dengue and malaria (present in other islands of this area of the Indian Ocean) as well as leptospirosis, a prevalent infection in Reunion Island [8]. If patients had travelled during the previous months in areas endemic for malaria, a blood smear was performed. If patients had travelled during the previous ten days, anti-dengue IgM and dengue RT-PCR assays were performed. Leptospirosis diagnosis using microagglutination and PCR assays were performed in the presence of clinical signs evocative of leptospirosis, such as jaundice.

Groups were compared using Chi-square or Fisher's exact test, when appropriate. Clinical and biological continuous parameters were compared with the Mann-Whitney test. Averages and standard deviation were calculated. A P value <0.05 was considered statistically significant.

In order to rationalize the choice of clinical and biological data for bivariate analysis, we looked for the sign with the highest correlation with a given group; the Yule coefficient was used to measure the strength of the link between the two variables (illness/sign). A Yule coefficient between 0.7 and 1 was considered as a very close link and 0.5–0.69 as a close link.

## Results

**Figure 1:**
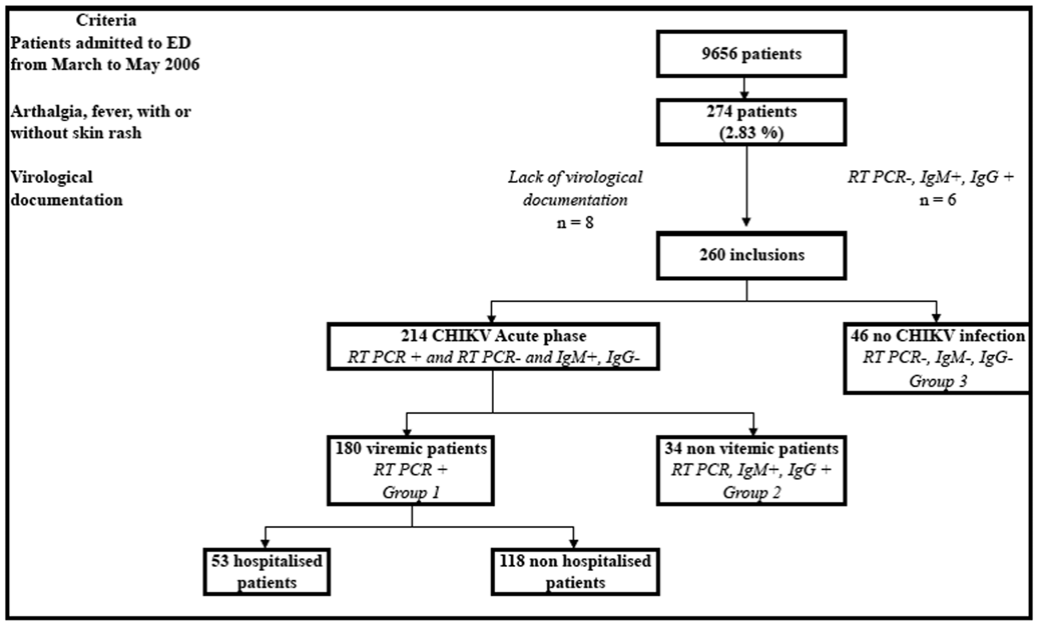
Patients entering the emergency department and displaying the clinical signs associated with Chikungunya.

During the study (1^st^ March 2006 - 31^st^ May 2006), 9,656 patients were admitted at the ED; 274 displayed febrile arthralgia and were included in the study (Figure 1). Because their virological data were missing, 8 patients had to be excluded. A CHIKV viremia was present in 180 patients (Group A1) and absent in 86 patients. Anti-CHIKV IgM were present in 40 of these 86 patients; among these 40 patients, 34 had negative anti-CHIKV IgG, and were in immediate post-viremic phase (Group A2). The remaining 46 patients had no CHIKV infection (Group B).

Among all patients admitted to the ED during the study, 2.21% were acutely infected by CHIKV and 1.86% were viremic. In acutely CHIKV-infected patients with fever and arthralgia (Group A), the positive predictive value of the presence of these two signs for CHIKV acute infection was 82.3%.

### Clinical features in Group A1 patients (Tables 1 to [Table pone-0007603-t002] [Table pone-0007603-t003] [Table pone-0007603-t004] 5)

In Group A1, the average age did not significantly differ between men and women. Co-morbidity was more common in women (72.6 vs. 56.3%; p<0.05), particularly high blood pressure (50.0 vs. 33.3%; p<0.05) and diabetes mellitus (33.3 vs. 16.6%; p<0.05). Co-morbidity was more frequent in patients of 65 years and over (97.4 vs. 39.4%; p<0.001). No case of leptospirosis, dengue or malaria was diagnosed in this group.

**Table 1:** Demographic data.

	Group A1 (n = 180)	Group A2 (n = 34)	Group B (n = 46)
**Age**	55.7±21.7	54.5±19,8	40.2±21.9 ***
**Range**	15–96	17–89	16–93
**Age ≥ 65 years old**	76 (42.2%)	17 (50.0%)	7 (15.2%) **
**Gender-ratio H/F**	1,14	0.62	0.92
**Comorbidity**	115 (63.9%)	24 (70.6%)	18 (39.1%) **
**- High blood pressure**	74 (41.1%)	15 (62.5%)	3 (16.7%)
**- Ischaemic heart disease**	23 (12.8%)	3 (12.5%)	0 (0.0%)
**- Cardiac failure**	17 (9.4%)	0 (0.0%)	1 (5.6%)
**- Diabetes mellitus**	44 (24.4%)	8 (33.3%)	3 (16.7%)
**- Dyslipidemia**	27 (15.0%)	3 (12.5%)	1 (5.6%)
**- Obesity**	12 (6.7%)	1 (2.9%)	1 (5.6%)
**- Pulmonary pathology**	21 (11.7%)	2 (5.9%)	2 (11.2%)
**- Renal failure**	15 (8.3%)	4 (16.7%)	0 (0.0%)
**- Cerebro-vascular accident**	14 (7.8%)	4 (16.7%)	1 (5.6%)
**- Epilepsy**	14 (7.8%)	0 (0.0%)	3 (16.7%)

***Group A1: RT PCR CHIKV positive patients. Group A2: RT PCR CHIKV negative, IgM anti CHIKV positive, IgG anti CHIKV negative patients. Group B: RT PCR CHIKV negative, IgM anti CHIKV negative, IgG anti CHIKV negative patients.***

***Group A1 vs. Group A2: ^¤¤¤^ p<0.001, ^¤¤^ p<0.01, ^¤^ p<0,05.***

***Group A1 vs. Group B:*** *** p<0.001, ** p<0.01, * p<0,05.

***Average, standard deviation, percentage in parenthesis are indicated.***

**Table 2 pone-0007603-t002:** Clinical signs.

	Group A1 (n = 180)	Group A2 (n = 34)	Group B (n = 46)
**Number of days between onset**			
**of symptoms and consultation**	1.8±1.9	**6.2±3.7^ ¤¤¤^**	2.9±2.5 **
**Range**	0–12	1–16	0–11
**Body temperature**	39.1±0,86	**37.9±0.6 ^¤¤¤^**	38.4±1.1 ***
**Body temperature ≥ 40°C**	35 (19.4%)	**0 (0.0%)^ ¤^**	6 (13.0%)
**Chill**	23 (12.8%)	3 (8.8%)	5 (10.9%)
**Myalgia**	106 (58.9%)	20 (58.8%)	31 (67.4%)
**Headache**	126 (70.0%)	20 (58.8%)	36 (78.3%)
**Skin rash**	86 (47.8%)	23 (67.7%)	13 (28.3%) *
**Adenopathy**	16 (8.9%)	**10 (29.4%)^ ¤¤^**	3 (6.5%)
**Pruritus**	25 (13.9%)	**14 (41.2%)^ ¤¤¤^**	7 (15.2%)
**Periarticular edemas**	46 (25.6%)	**15 (44.1%)^ ¤^**	5 (10.9%)
**Conjonctival hyperhemia**	41(22.8%)	6 (17.7%)	6 (13.0%)
**Digestive signs**	114 (63.3%)	**29 (85.3%)^ ¤^**	37 (80.4%) *
**- Nausea**	67 (58.8%)	20 (69.0%)	27 (73.0%)
**- Vomiting**	49 (43.0%)	11 (37.9%)	22 (59.5%)
**- Diarrhea**	33 (28.9%)	11 (37.9%)	13 (35.1%)
**- Anorexia**	80 (70.2%)	25 (86.2%)	23 (62.2%)
**- Dysgueusia**	46 (40.4%)	17 (58.6%)	13 (35.1%)
**- Abdominal pain**	36 (31.6%)	5 (17.2%)	15 (40.5%)
**Bleeding** (gingivorrhagia, epistaxis)	2 (1.1%)	**3 (8.8%)^ ¤^**	1 (2.2%)
**Asthenia**	159 (88.3%)	31 (91.2%)	33 (71.7%) *
**Lipothymia, vertigo**	20 (11.1%)	4 (11.8%)	5 (10.9%)
**Fainting**	9 (5.0%)	0 (0.0%)	2 (4.3%)
**Mental confusion**	12 (6.7%)	3(8.8%)	1 (2.2%)
**Attention deficit disorder**	11 (6.1%)	2 (5.9%)	0 (0.0%)
**Dry cough**	14 (7.8%)	0 (0.0%)	5 (10.9%)

***Group A1: RT PCR CHIKV positive patients. Group A2: RT PCR CHIKV negative, IgM anti CHIKV positive, IgG anti CHIKV negative patients. Group B: RT PCR CHIKV negative, IgM anti CHIKV negative, IgG anti CHIKV negative patients.***

***Group A1 vs Group A2: ^¤¤¤^ p<0.001, ^¤¤^ p<0.01, ^¤^ p<0,05.***

***Group A1 vs. Group B:*** *** p<0.001, ** p<0.01, * p<0,05.

***Average, standard deviation, percentage in parenthesis are indicated.***

**Table 3 pone-0007603-t003:** Arthralgia location.

	Group A1 (n = 171)	Group A2 (n = 31)	Group B (n = 42)
**Upper limbs**	155 (90.6%)	26 (83.9%)	36 (85.7%)
**- Interphalangian joints**	90 (52.6%)	16 (51.6%)	17 (40.5%)
**- Metacarpo-phalangeal joints**	81 (47.4%)	19 (62.2%)	12 (28.6%)
**- Wrists**	121 (59.7%)	23 (74.2%)	21 (50.0%) *
**- Elbows**	81 (47.4%)	14 (45.2%)	12 (28.6%)
**- Shoulders**	86 (50.3%)	17 (54.8%)	23 (57.1%)
**Lower limbs**	161 (94.1%)	29 (93.4%)	40 (95.2%)
**- Feet**	83 (48.5%)	18 (58.1%)	10 (23.8%) **
**- Ankles**	130 (76.0%)	26 (83.9%)	28 (66.7%)
**- Knees**	130 (76.0%)	25 (80.6%)	27 (64.3%)
**- Hips**	29 (17.0%)	2 (6.5%)	7 (16.7%)
**Rachis**	79 (46.2%)	14 (45.2%)	25 (59.5%)
**Sternocostal joints**	37 (21.6%)	5 (16.1%)	11 (26.2%)
**Temporomandibular**	3 (1.8%)	1 (3.2%)	1 (2.4%)
**Large joints**	169 (98.8%)	30 (96.8%)	40 (95.2%)
**Small joints**	128 (74.8%)	23 (74.2%)	26 (61.9%)

***Group A1: RT PCR CHIKV positive patients. Group A2: RT PCR CHIKV negative, IgM anti CHIKV positive, IgG anti CHIKV negative patients. Group B: RT PCR CHIKV negative, IgM anti CHIKV negative, IgG anti CHIKV negative patients.***

***Group A1 vs. Group A2: ^¤¤¤^ p<0.001, ^¤¤^ p<0.01, ^¤^ p<0,05.***

***Group A1 vs. Group B:*** *** p<0.001, ** p<0.01, * p<0,05.

***Average, standard deviation, percentage in parenthesis are indicated.***

**Table 4 pone-0007603-t004:** Periarticular oedemas.

	Group A1 (n = 180)	Group A2 (n = 34)	Group B (n = 46)
**Number**	46 (25.6%)	15 (44.1%)·	5 (10.9%)
**Location**			
**- Fingers**	22 (47.8%)	6 (40.0%)	3 (60.0%)
**- Wrists**	12 (26.1%)	5 (33.3%)	2 (40.0%)
**- Elbows**	1 (2.2%)	0 (0.0%)	0 (0.0%)
**- Feet/Toes**	10 (21.7%)	3 (20.0%)	1 (20.0%)
**- Ankles**	31 (67.4%)	8 (53.3%)	1 (20.0%)
**- Knees**	4 (8.7%)	0 (0.0%)	0 (0.0%)

***Group A1: RT PCR CHIKV positive patients. Group A2: RT PCR CHIKV negative, IgM anti CHIKV positive, IgG anti CHIKV negative patients. Group B: RT PCR CHIKV negative, IgM anti CHIKV negative, IgG anti CHIKV negative patients.***

***Group A1 vs. Group A2: ^¤¤¤^ p<0.001, ^¤¤^ p<0.01, ^¤^ p<0,05.***

***Group A1 vs. Group B:*** *** p<0.001, ** p<0.01, * p<0,05.

***Average, standard deviation, percentage in parenthesis are indicated.***

**Table 5:** Cutaneomucosal signs.

	Group A1 (n = 180)	Group A2 (n = 34)	Group B (n = 46)
**Exanthem**	86 (47.8%)	23 (67.7%)	13 (28.3%) *
**Diffuse exanthem**	16 (8.9%)	**11 (32.4%)^ ¤¤¤^**	1 (2.2%)
**Exanthem with healthy skin intervals**	70 (38.9%)	**12 (35.3%)^ ¤¤¤^**	12 (26.1%)
**- Topography**			
**- Face**	26 (37.1%)	3 (25.0%)	4 (30.8%)
**- Neck**	26 (37.1%)	3 (25.0%)	2 (15.4%)
**- Trunk**	45 (64.3%)	8 (66.7%)	7 (53.9%)
**- Arms**	28 (40.0%)	5 (41.7%)	4 (30.8%)
**- Forearms**	19 (27.1%)	3 (25.0%)	1 (7.7%)
**- Hands**	6 (8.6%)	0 (0.0%)	1 (7.7%)
**- Thighs**	9 (12.9%)	**7 (58.3%)^ ¤¤^**	4 (30.8%)
**- Legs**	18 (25.7%)	5 (41.7%)	2 (15.4%)
**- Feet**	5 (7.1%)	0 (0.0%)	1 (7.7%)
**Purpura**	4 (2.2%)	3 (8.8%)	0 (0.0%)
**Vesicles/bubbles**	3 (1.7%)	0 (0.0%)	0 (0.0%)
**Pruritus**	25 (13,9%)	**14 (41.2^%) ¤¤¤^**	7 (15.2%)
**Pruritus whitout skin lesions**	8 (4.4%)	2 (5.9%)	6 (13.0%) *
**Cutaneous hyperesthesia**	7 (3.9%)	0 (0.0%)	0 (0.0%)
**Conjunctival hyperhemia**	41 (22.8%)	6 (17.7%)	6 (13.0%)
**Mouth ulcers**	0 (0.0%)	1 (2.9%)	0 (0.0%)
**Pharyngitis**	2 (1.1%)	0 (0.0%)	1 (7.7%)

***Group A1: RT PCR CHIKV positive patients. Group A2: RT PCR CHIKV negative, IgM anti CHIKV positive, IgG anti CHIKV negative patients. Group B: RT PCR CHIKV negative, IgM anti CHIKV negative, IgG anti CHIKV negative patients.***

***Group A1 vs. Group A2: ^¤¤¤^ p<0.001, ^¤¤^ p<0.01, ^¤^ p<0,05.***

***Group A1 vs. Group B:*** *** p<0.001, ** p<0.01, * p<0,05.

***Average, standard deviation, percentage in parenthesis are indicated.***

Most patients presented to the ED during the first 3 days after the onset of symptoms: 18.3% on the 1^st^ day, 41.1% on the 2^nd^ day, 18.3% on the 3^rd^ day, and 10.0% on 4^th^ day. The onset of symptoms was often described as abrupt, without prodromic phase. The highest body temperatures were recorded in patients who consulted within the 2 days that followed the onset of symptoms (1^st^ day: 39.3±0.8°C; 2^nd^ day: 39.2±0.9°C; 3^rd^ day: 38.9±0.8°C; 4^th^ day: 38.9±0.9°C; 5^th^ day: 38.4±0.8°C; 1^st^ day-2^nd^ day vs. 5^th^ day: p<0.05).

Data on osteoligamentous pain were available for 171 patients. The 9 remaining patients were disoriented, either acutely or chronically in the context of Alzheimer's disease, and although they complained from arthralgia, they were unable to describe them further. The joint symptoms were most often characterized by bilateral and symmetrical arthralgia (n = 165; 96.5%). In few cases, only 2 to 3 joints were affected (n = 4; 2.3%), and only two patients (1.2%) suffered from monoarthralgia. Impairment of the finger joints was more frequent in women than men (66.7 vs. 47.9%; p<0.01). Nineteen patients (10.5%) suffered from pain along previous bone fractures or ligaments injuries (tendinopathy, Achilles' tendon rupture) as well as increased arthrosic pain. Clinical examination revealed pain along ligament insertions (pubalgia, sternocleidomastoid and occipital insertions) in 3 patients (1.6%). Talalgia were observed in 24 patients (14.0%). Joint inflammatory signs were rare (n = 2; 1.1%) and peri-articular swellings were more frequent in women than men (36.9 vs. 15.6%; p<0.01).

Exanthema was either morbilliform, roseola-like, or more rarely maculopapular. An erysipela aspect of the lower limbs was reported in two patients (1.1%). Hemorrhagic signs were extremely rare and were not associated with clotting abnormalities or major thrombocytopenia. Uncommon neurological symptoms included cutaneous dysesthesia (n = 7; 3.9%) mostly of the sole of the feet, hallucinations (n = 2; 1.1%), paresthesia in the ulnar nerve territory (n = 1; 0.6%). Three patients (1.7%) presented with convulsion: one patient was epileptic, another one exhibited frequent comitial crises as stroke sequelae, and the last one suffered from alcohol withdrawal.

During the viremic phase of CHIKV infection, 3 patients (1.7%) decompensated a pre-existing cardiac insufficiency. Chest pain was associated with pericarditis (n = 2; 1.1%), acute coronary syndrome (n = 4; 2.2%), and myocarditis (n = 2; 1.1%). The registered cardiac arrhythmias (atrial fibrillation n = 4, ventricular extra systole n = 2) were pre-existing conditions. Dyspnoea was observed in 17 patients (9.4%), who were older than the average age (71 year-old) and affected with respiratory or cardiac disorders or complications (acute pneumopathy). Two patients of 15 and 18 year-old complained from abdominal pain and displayed clinical signs of mesenteric lymphadenitis associated with peripheral adenopathies.

### Biological parameters in Group A1 patients (Table 6)

The blood platelet counts were lower for patients examined on 5^th^ day (134,143±33,354/mm^3^) than for patients examined on 1^st^ day (195,813±51,859/mm^3^; p<0.01) or 2^nd^ day (183,068±53,996/mm^3^; p<0.05). During the viremic phase, the average blood lymphocytes values were under the normal range, without significant difference compared to the onset of symptoms.

**Table 6:** Biological parameters.

	Groupe A1 (n = 180)	Groupe A2 (n = 34)	Groupe B (n = 46)
**Platelets**	174288±55962	173735±62235	193444±92057
(150000–500000/mm^3^)#	(38000–355000)	(46000–330000)	(26000–579000)
**Leucocytes**	5,431±2,139	5,199±2,554	7,429±3,640 ***
(4500–13500/mm^3^)#	(1,900–14,800)	(1,400–12,400)	(1,700–16,600)
**Lymphocytes**	608±314	**1,090±481^ ¤ ¤¤^**	1,141±747 ***
(1000–4000/mm^3^)#	(100–2,360)	(300–2,500)	(200–3,400)
**Urea**	6.8±4.9	6.1±3.2	5.8±4.401
(2.5–7.5 mmol/L)#	(1.6–37.0)	(2.6–16.0)	(2.3–27.6)
**Creatinine**	111,1±73,1	104.2±100.9	89.3±48.4 **
(50–100 mmol/L)#	(40.0–604.0)	(53.0–661.0)	(35.0–328.0)
**CRP**	55.9±50.4	**30.5±49.7^¤¤¤^**	73.3±108.5
(<2.5 mg/L)#	(0.0–351.0)	0.0–267.0)	(0.0–492.0)
**Calcemia**	2.28±0.14	2.31±0.12	2.33±0.15 **
(2.25–2.65 mmol/L)#	(1.8–2.7)	(2.05–2.66)	(1.96–2.75)
**ASAT**	55.0±164.0	53.2±42.3	333.2±1952.8
(<45 UI/L)#	(12.0–2177.0)	(16.0–195.0)	(13.0–13140.0)
**ALAT**	35.2±89.7	**39.5±29.9^ ¤^**	122.9±651.4
(<65 UI/L)#	(7.0–1189.0)	(11.0–125.0)	(8.0–4394.0)
**CK**	320.6±794.3	334.6±630.7	291.6±542.7
(<210 UI/L)#	(22.0–7608.0)	(25.0–2901.0)	(28.0–3209.0)
**Lipase**	42.5±29.3	42.3±19.9	32.0±26.2 **
(<60 UI/L)#	(9.0–202.0)	(15.0–101.1)	(9.0–180.0)

***Group A1: RT PCR CHIKV positive patients. Group A2: RT PCR CHIKV negative, IgM anti CHIKV positive, IgG anti CHIKV negative patients. Group B: RT PCR CHIKV negative, IgM anti CHIKV negative, IgG anti CHIKV negative patients.***

***Group A1 vs. Group A2: ^¤¤¤^ p<0.001, ^¤¤^ p<0.01, ^¤^ p<0,05.***

***Group A1 vs. Group B:*** *** p<0.001, ** p<0.01, * p<0,05.

***Average, standard deviation, percentage in parenthesis are indicated.***

^#^ ***Physiological ranges.***

A significant difference for the blood biological parameters between genders was found for platelets (men 161,298±49,050/mm^3^; women 189,000±59,838/mm^3^; p<0.01), creatinine (men 117.4±60.9 mmol/L; women 104.1±84.5 mmol/L; p<0.001), alanine aminotransferase (men 31.8±22.4 UI/L; women 38.8±128.4 UI/L; p<0.01) and creatine phosphokinase (men 361±874 UI/L; women 275.0±696.0 UI/L; p<0.01).

Viral load was significantly higher in patients with comorbidity than those without (2.29 10^6^±11.9 10^6^ vs. 3.04 10^5^±7.48 10^5^ copies/mL; p<0.05). Viral load was also significantly higher in patients 65 year-old and older than in patients less than 65 year-old (3.86 10^5^±11.99 10^5^ vs. 3.19 10^6^±14.55 10^6^ copies/mL; p<0.001).

### Hospitalized patients in Group A

Sixty-nine patients (32.2%) were hospitalized. Two patients (83 and 73 year-old) died while they were hospitalized because of a nosocomial *Pseudomonas aeruginosa* associated pneumopathy with septicemia in one case and a coronary insufficiency in the other. The hospitalized patients were older (p<0.001) and had higher frequency of comorbidity (p<0.001) than the non-hospitalized ones, these two factors being linked (p<0.01). Clinically, the hospitalized patients did not differ from the non-hospitalized ones except for periarticular oedemas, metacarpo-phalangeal joints and feet arthralgia (p<0.05). The blood levels of urea, C-reactive protein, creatinine phosphokinase, ASAT were significantly higher (p<0.01) for hospitalized patients. In Group A1, the viral load was significantly higher in hospitalized patients than in non-hospitalized patients (p<0.05).

Age over 65 was the factor most associated with severity (Yule coefficient 0.78, sensitivity 74.6%, specificity 73.0%, positive predictive value 57.9% negative predictive value 85.6%).

### Clinical parameters in CHIKV uninfected patients with febrile arthralgia (Group B)

The diagnoses in Group B differed, depending on whether the patients had to be hospitalized or not. For non-hospitalized patients (n = 33), suspicion of CHIKV infection was the diagnosis most frequently mentioned but not confirmed biologically later on (n = 25) (no alternative diagnosis was later obtained); other diagnoses were malaria (n = 2), pyelonephritis (n = 4), gastroenteritis (n = 1), pharyngitis (n = 1). Among hospitalized patients (n = 13), diagnoses were pneumopathy (n = 4), pyelonephritis (n = 3), leptospirosis (n = 2), gastroenteritis (n = 1), migraine (n = 1), fulminant hepatitis of unknown origin (n = 1), microcrystalline arthropathy (n = 1).

### Comparison between groups of patients

*Acutely CHIKV-infected patients (Group A) vs. patients without CHIKV infection (Group B)* – Clinical data are reported in the tables 1 to [Table pone-0007603-t002] [Table pone-0007603-t003] [Table pone-0007603-t004] 5. A total leukocyte blood count lower than 7.400/mm^3^ was closely associated with CHIKV infection (Yule coefficient 0.63, sensitivity 88.7%, specificity 36.4%, positive predictive value 80.4% negative predictive value 52,2%).

*Viremic (Group A1) vs. post-viremic (Group A2) CHIKV infected patients* – During the CHIKV acute period, there was a significant difference between viremic (Group A1) and post-viremic (Group A2) (Tables 2, 5 & 6). Lymphopenia (<1,000/mm^3^) was very closely associated with Group A1 (Yule coefficient 0.82, sensitivity 86.1%, specificity 61.8%, positive predictive value 92.3%, negative predictive value 45.7%).

*Viremic acute phase CHIKV infected patients (Group A1) vs. patients without CHIKV infection (Group B)* – Significant clinical and biological differences between group A1 and group B are listed in the Tables 1, 2 & 6. Lymphopenia (<1,000/mm^3^) was very closely related to Group A1 (Yule coefficient 0.72, sensitivity 86.1%, specificity 50.0%, positive predictive value 87.1% negative predictive value 47.9%).

## Discussion

We carried out a prospective study of the clinical and biological features characterizing the acute viremic and post-viremic phases of human Chikungunnya infection in patients referred to the ED for febrile athralgia. The patients without Chikungunya entering the ED with fever and arthralgia during the study were used as a control group. The monthly numbers of newly admitted patients with CHIKV viremia in our study were consistent with the estimated incidence in La Réunion [4] (March 2006 32,500, April 11,800, May 5,300). The percentages of viremic patients were stable along the study and represented 0.5% of the total estimated new cases in the Island in March, 0.45% in April and 0.45% in May. Consequently, the newly admitted viremic patients at the ED are representative of the epidemic. Yet, this study does not take into account asymptomatic infections, but they are thought to represent an extremely low proportion (5%) [3] when compared to other arbovirosis such as dengue (80%) [9], [10].

To our knowledge, this is the first prospective study on the clinical and biological aspects of acute Chikungunya infections in adults with differentiation between the viremic and non-viremic phases during an outbreak. Previously published clinical and biological data were obtained from retrospective analyses, usually in the absence of virological confirmation of acute infection. Several studies have used anti-CHIKV IgM as an acute phase criterion but they are still present in more than 50% of patients a year after the onset of the disease [5]. Simon *et al*. conducted a prospective observational clinical study during the early stages of Chikungunya infections (within 10 days of the disease onset) but did not differentiate between the viremic and non-viremic phases [11]. In addition, they included in their study data from a retrospective questionnaire completed by patients examined 10 days after the onset of symptoms. Recent retrospective studies reviewing cases that occurred in the Indian Ocean were published. Taubitz *et al*. described clinical and biological results for 20 travellers admitted in their department 2 to 73 days after the onset of symptoms [12]. Borgherini *et al*. published early biological and clinical features for 157 adult patients during the outbreak on Reunion Island [13]. Borgherini's study displays the biases inherent to retrospective surveys. In particular, the patient's selection and data collection processes did not include frequent clinical signs such as myalgia. This study also differs from previous works since its patients constitute an unbiased representative sample of the population enrolling at the ED over a continuous three-month period, and describes the clinical and biological features of the viremic and post-viremic phases of the acute CHIKV infection, as assessed by RT-PCR and IgM assays in 216 consecutive patients.

Moreover, most of these retrospective studies were carried out in countries where co-infections with arboviruses such as Dengue and Yellow-fever viruses are likely to occur [14]. We also searched for co-infections. No case of dengue fever was diagnosed in our study, and no cases of dengue fever or other arbovirosis was reported in 2006 in La Réunion, therefore reinforcing the specificity of our findings. Malaria and leptospirosis were not diagnosed in our patients infected with CHIKV. We found rare and benign hemorrhagic signs, in contrast to what previously reported in India and Southeast Asia —areas where Dengue is endemo-epidemic— where CHIKV was incriminated in haemorrhagic fever outbreaks [15]. Hemorrhagic signs in other studies among adults were probably attributable to co-infection, especially with dengue or yellow fever viruses, even if Asian CHIKV genotypes differ from the one responsible for the Indian Ocean outbreak, which is of East African origin [16]. During the outbreak in La Réunion, hemorrhagic signs were described in our prospective study of neonatal Chikungunya infection [17] and thrombocytopenia and haemostasis troubles were also common in the neonatal context.

Since 1953, the classical clinical features of acute Chikungunya associate the triad fever, arthralgia and inconstant skin rash [18]. We showed here that fever is concomitant of viremia, with the highest temperatures in the 2 days that follow the onset of the symptoms.

Arthralgia is key to the clinical diagnosis of acute CHIKV infection. However, forms with secondary arthralgia or without arthralgia have been described in Asian studies [19]. In a random sample of the population of La Réunion during the post-epidemic phase, 9.7% of proven Chikungunya cases (*i.e*. with a positive serology) reported no arthralgia, including the 5% asymptomatic forms [3]. In our study, the presence of arthralgia was mandatory for inclusion, and asymptomatic forms were obviously not referred to the ED. CHIKV-associated arthralgia have not been characterized in detail so far [20], [21]. While in most cases, bilateral and symmetrical polyarthralgia were reported (although sometimes more intense on one side), we observed two cases of monoarticular arthralgia of the ankle. We recorded rare locations of arthralgia, such as of the temporo-mandibular joint, and forms that had never been reported in previous descriptions such as chondrocostal arthralgia, affecting up to 20% of the patients, and hip arthralgia (17%). CHIKV also appeared to revive pain along former fractures lines and ligament injuries, and clinical examination revealed entesopathies and talalgia, which had never been described previously.

The frequency of skin rash ranged from 20% to 85% [18], [22]. In our study, about half of the patients presented a skin rash, generally arising during the first three days of infection. Our study matches former studies in the location and aspect of the skin lesions but differs by the existence of vesicular and bullous forms as well as erysipela-like lesions of the lower limbs. By mean of RT-PCR, we could document the specificity of these skin manifestations by amplifying the viral genome within this vesicular fluid (our unpublished data).

Our study illustrates the variety of the other clinical manifestations that can be associated with CHIKV infection. Yet, these symptoms are non-specific as they can be present in a number of other viral infections. Some of these clinical symptoms may also correspond to decompensation of underlying conditions and to iatrogenic effects (e.g. paracetamol and hepatitis, non-steroidal anti-inflammatory and kidney failure).

Severe clinical forms with neurological [23], [24] and cardiac involvements [25], [26] have also been reported in the literature. In 1973, Obeyesekere [25] described the first cases of acute pericarditis and myocardiopathy associated with CHIKV, based on serology. Thus, CHIKV may, as other viruses, trigger pericarditis and myocarditis. Lemant *et al*. [27], in a retrospective survey of CHIKV-infected patients admitted to intensive care, reported a case of fatal fulminant myocarditis for which analysis of post-mortem myocardial tissue samples pathological examination demonstrated the presence of cytoplasmic viral inclusions in myocytes. Cardiac complications and myocarditis have also been described in infected newborns on the Island of La Réunion [17], [28].

Neurological symptoms (vertigo or confusion) observed in our study may be attributable to fever and dehydration. However, a neurotropism of CHIKV has several times been suspected [14], [29], [30]. Mazaud [30] reported a case of acute encephalitic syndrome, and Deller [29] described the onset of vertigo and a vestibular syndrome. In India, Carey [14] observed important neurological after-effects. In La Réunion, severe cases of meningo-encephalopathy among adults [27] and newborns [28], and acute polyradiculoneuritis were also attributed to CHIKV [24]. Consistent with these clinical observations, we have shown, in an animal model for Chikungunya, that CHIKV may disseminate to the central nervous system, and infect the meninges and the ependymal tissue [31].

Mazaud [30] observed biological similarities between dengue and chikungunya. Indeed, it was reported that these two arbovirosis are associated with brief leukoneutropenia (3 to 4 days) and thrombocytopenia [30]. According to this author, leukoneutropenia and thrombocytopenia have great orientation value in favour of these two arboviroses. In other studies, no leukopenia was reported [19]. In contrast to these retrospective studies in which the differential diagnosis between dengue and chikungunya could not be precisely assessed, we found in our series that 38% of patients were leukopenic and that in Group A1, lymphopenia dominated and was very closely associated with viremia; for 85% of the patients lymphocyte count was lower or equal to 1,000/mm^3^ and for 50% of the patients the level was lower than 500/mm^3^. Thrombocytopenia was found in 42% of the patients, but to moderate levels, between 100,000 and 150,000/mm^3^.

Cases of severe acute hepatitis occurring during the CHIKV infection have already been described in La Réunion. While it was suggested that this virus may target the liver –a finding confirmed in our animal model in the first hours of infection–, acute hepatitis seems to be mainly promoted by chronic ethylism, denutrition and paracetamol toxicity [32]. The serum ASAT level was increased for 32% of the patients as opposed to only 7% for the ALAT. These results suggest that the increase in the serum level of ASAT was partially related to muscle injury, with 60% of the patients with increased ASAT also presented a rhabdomyolysis.

When using the two cardinal clinical signs in acute phase, fever and arthralgia, for the diagnosis, we found a specificity of 99,6% and a positive predictive value of 84.6%. Leucopenia and lymphopenia could be used to aid in the diagnosis of CHIKV infection in acute phase, but not for formal diagnosis. The positive predictive values for these parameters, found in our work, are inappropriate. Indeed, the high number of CHIKV-infected patients compared to controls tends to artificially raise the positive predictive values and decrease the negative predictive value. So, leucopenia/lymphopenia values are suggested as only one parameter that could be a part of a diagnosis algorithm. Other mosquito-transmitted viruses with overlapping geographic distributions, specifically Dengue Fever, can have similar hematologic abnormalities. However because of the risk of transmission of the Chikungunya fever in European countries free of Dengue Fever, these hematologic abnormalities are of interest and value.

Factors influencing disease severity are dominated by age and comorbidities. However, these two criteria are strongly linked. Among viremic patients, age under 65 was an excellent predictor of non-severity (severity negative predictive value 85.6%), with only functional impotence due to lower limbs pain and inability to walk leading to hospitalization in this group.

Little is known about the pathophysiology of CHIKV infection, in contrast to better characterized alphaviruses such as Sindbis and Ross River viruses. Sourisseau *et al*. [33] have reported that CHIKV replicates in various human cultured cells, such as epithelial and endothelial cells, primary fibroblasts and, to a lesser extent, macrophages. CHIKV replication induces a significant cytopathic effect, and may also lead to apoptosis. CHIKV replication is significantly inhibited by type I IFNs, suggesting a prominent role of the innate immune system in the control of the infection and the rapid decline of viremia before the appearance of neutralizing antibodies during the acute phase of infection. It has also been reported that CHIKV may also infect muscle satellite cells in humans [34]. In a mouse model of chikungunya, we have shown that fibroblasts of tissues in which seats symptoms in humans (muscles, joints, skin), are the target cells of CHIKV [31], these findings matching viral tropism in human as assessed by CHIKV immunolabeling of infected human tissues. The infection of muscles and joints, which contain nerve endings may account for the nociceptive pain observed in the course of human acute infection. The functional neurological manifestations complicating the course of acute CHIKV infection may reflect CHIKV dissemination to the central nervous system, consistent with the positive CHIKV RT-PCR in the cerebrospinal fluid of these patients and the observation that CHIKV infect the choroid plexuses, meninges and the ependyma in the mouse animal model [31].

The worldwide distribution of *Aedes albopictus* and the adaptation of the virus to this vector [16], [35] may cause the emergence and spread of epidemic CHIKV in North and South America and in European countries, as illustrated recently in Ital [36]. In addition to the basic original information it provides on this little known disease, this study should be of help to clinicians confronted to a CHIKV outbreak, for clinical and logistic management of acutely infected patients presenting at the emergency room.
